# Development and validation of the Revised Epistemic Trust, Mistrust and Credulity Questionnaire (ETMCQ-R)

**DOI:** 10.1192/bjo.2025.10813

**Published:** 2025-09-01

**Authors:** Chloe Campbell, Henry Delamain, Rob Saunders, Michal Tanzer, Alberto Milesi, Tobias Nolte, Elizabeth Allison, Patrick Luyten, Peter Fonagy

**Affiliations:** Research Department of Clinical, Educational and Health Psychology, University College London, London, UK; Anna Freud, London, UK; CORE Data Lab, Centre for Outcomes Research and Effectiveness (CORE), Research Department of Clinical, Educational and Health Psychology, University College London, London, UK; Department of Psychology, University of Milano-Bicocca, Milan, Italy; Faculty of Psychology and Educational Sciences, University of Leuven, Leuven, Belgium

**Keywords:** Epistemic trust, mentalising, psychopathology, childhood adversity, developmental psychopathology

## Abstract

**Background:**

It has been argued that disruptions to epistemic trust are implicated in psychopathology; however, this requires empirical testing, and an existing scale evaluating epistemic trust, the Epistemic Trust, Mistrust and Credulity Questionnaire (ETMCQ), requires improvement.

**Aims:**

This study tested a revised version of the Epistemic Trust, Mistrust and Credulity Questionnaire (the ETMCQ-R), examining the strength of associations between the updated scale and mental health symptoms, epistemic vice, psychological resilience, perceived social support, attachment style, history of childhood adversity and an experimental measure of trust, and epistemic stance as a mediator between adversity and psychopathology.

**Method:**

Using an online survey design, 525 participants completed the ETMCQ-R alongside other measures. Exploratory and confirmatory factor analyses were conducted to assess the structure of the ETMCQ-R and correlational and mediational analyses were used to further assess validity of the measure.

**Results:**

The ETMCQ-R possesses greater model fit and a stronger three-factor structure (Trust, Mistrust and Credulity) compared with the ETMCQ. Significant negative correlations were identified between Trust (*r* = −0.12) and higher scores on global psychopathology severity, while Mistrust (*r* = 0.41) and Credulity (*r* = 0.36) showed positive correlations. Trust negatively correlated with borderline features (*r* = −0.10), whereas Mistrust and Credulity positively correlated (*r* = 0.54 and *r* = 0.48, respectively). Mistrust and credulity partially mediated the relationship between childhood adversity and psychopathology, with stronger mediation effects for borderline features than general psychopathology.

**Conclusion:**

The study demonstrated strong psychometric properties of the ETMCQ-R, and further analyses indicate the three factors are differentially related to wider domains of socio-emotional functioning.

The application of the concept of epistemic trust, and epistemic disruption, to understanding psychopathology has garnered increasing research interest over the past 10 years. According to this framework, epistemic disruption has been characterised by epistemic mistrust, an increased wariness of information providers, and/or epistemic credulity, a propensity to accept knowledge even from potentially unreliable sources without question. Epistemic trust, meanwhile, is defined as openness to being influenced by dependable social sources.^1^ It has been argued that disruptions to epistemic trust might signal a broader vulnerability to psychopathology.^2^

The epistemic trust framework described here is an evolutionarily informed developmental model, which proposes that humans are primed to adopt a disrupted epistemic position in response to negative social stimuli, and adverse childhood experiences (ACEs) in particular, as such exposure generates an expectation that others cannot be regarded as reliable or benignly motivated sources of knowledge.^2^ It has further been argued that disruption in the capacity for epistemic trust may be one of the mechanisms by which adversity renders an individual more vulnerable to psychopathology: a reduced ability to trust others as social sources of information obstructs social learning and interrelational mentalising, which impairs healthy adaptation and resilience.^2^ Thus, it has been argued that epistemic disruption may be particularly associated with more complex and severe mental health conditions that are characterised by interpersonal difficulties and compromised mentalising capacity.^2^

Recent empirical studies, which have included behavioural experiments and self-report techniques,^3,4^ have started to evaluate this theory. The Epistemic Trust, Mistrust and Credulity Questionnaire (ETMCQ), a validated tool, assesses an individual’s openness to new knowledge (their epistemic stance) through three dimensions: epistemic trust, mistrust (increased wariness of information providers) and credulity. Research using the ETMCQ has shown that epistemic disturbances, characterised by high levels of mistrust and/or credulity, are connected to a greater risk of psychopathological symptoms, insecure attachment patterns, challenges in mentalising and a higher frequency of negative experiences in childhood. Both mistrust and credulity have been found to partially mediate the relationship between early adversity and symptoms of mental health disorder,^1^ a finding that has been replicated in the context of the relationship between adversity and post-traumatic stress disorder (PTSD) and complex PTSD.^5^

The ETMCQ has been adapted and validated in French,^6^ German,^5,7^ Italian,^8–10^ Persian,^11,12^ Serbian^13^ and Argentine Spanish.^14^ These studies have so far largely involved community samples, mostly of adults.^5–7,10–18^ Some studies have used adolescent or emerging adult community samples.^8,19–25^ These studies found a broadly similar factor structure to the English version, although some items seem to perform differently in other languages, leading to minor modifications. Across these cross-sectional studies, similar observations were made for associations between the ETMCQ and key developmental and psychological factors such as childhood trauma, attachment style, mentalising capacity and symptoms of mental health disorder as in the original ETMCQ study.^1^

In addition, in support of the position that epistemic stance (i.e. an individual’s level of trust, mistrust and/or credulity) mediates the relationship between adversity and vulnerability, a study of the relationship between ACEs and complex PTSD found epistemic stance to be a relevant mediator.^5^ Epistemic stance has also been found to be a mediator between childhood traumatic experiences and psychopathology in samples of community adults^6^ and community adolescents.^25^

Evidence is also emerging for the use of the ETMCQ in clinical samples.^7,26,27^ This evidence suggests that epistemic stance may contribute to the response to therapy,^26–28^ as suggested by the theoretical framework.^2^ For example, in a naturalistic longitudinal observational study with 771 participants in psychosomatic in-patient rehabilitation, Riedl et al,^26^ using the German translation of the ETMCQ, found that the participants whose psychological distress showed the greatest improvement had significantly improved epistemic trust and reduced epistemic mistrust, whereas those who showed the least alleviation of their symptoms reported a significant increase in epistemic mistrust and credulity.

Despite the valuable findings arising from the replication and validation of the original version of the ETMCQ, three significant challenges have emerged, suggesting the need for a revised scale. The first challenge is that, across studies, the Trust subscale of the ETMCQ shows weaker correlational strength compared with the Mistrust and Credulity subscales. These latter dimensions have demonstrated stronger links with factors such as exposure to childhood adversity and psychopathology, whereas Trust has not been strongly correlated with either reduced adversity or better mental health outcomes.^7,8,26^ This finding across various studies has led some researchers to suggest the possible removal of the Trust subscale from the instrument.^12^ Second, the factor loading of certain items was poor, and in one study items were observed to load on to an unexpected factor.^8^ The third challenge concerns reliability, specifically, that some items were found to be marginally or insufficiently reliable.^5,8^ These issues collectively indicate that the ETMCQ might benefit from revision, especially regarding the content of its items. This paper thus reports the outcomes of a comprehensive pre-registered validation study that examined the psychometric properties of a revised version of the ETMCQ (the ETMCQ-R).

In addition to testing the psychometric properties of the ETMCQ-R, we sought to further test the constructs of epistemic trust, mistrust and credulity in relation to other variables. This was undertaken partly with a view to replicating the original study, but in addition we sought to develop our understanding of epistemic stance and socio-emotional and social-cognitive functioning. In particular, in order to explore and define more clearly what we are measuring when we are seeking to assess epistemic trust as we have conceptualised it, we sought to test the associations between the ETMCQ-R and a task involving assessments of facial trustworthiness and a questionnaire on ‘epistemic vice’. The assessment of facial trustworthiness, using a well-established task developed by Todorov et al.^29^ involving exposure to a human face alongside the question ‘Do you trust this face?’, was chosen as a measure of more general interpersonal trust compared with the ETMCQ-R’s emphasis on trust in the social communication of information. Meanwhile, the Epistemic Vice Scale, which is a recently developed measure of aspects of epistemic trust, seeks to assess potential ways of thinking that obstruct the acquisition and sharing of knowledge.^30^ Comprising two subscales – tendency to rigidity and tendency to indifference about the truth – this scale makes a valuable contribution to measuring epistemic stance as a cognitive trait. We included this scale as we wanted to test our socially nested measure of epistemic trust in relation to more cognitive expressions of epistemic trust. In a further replication of our original study, and in order to further test one of the key premises of the developmental framework, we undertook mediation analyses to explore the role of epistemic stance, as measured by the ETMCQ-R, as a mediator between adversity in childhood and current mental health outcomes, which we measured using a general scale of psychopathology (the Brief Symptom Inventory (BSI)) and, more specifically, the presence of symptoms of borderline personality disorder (BPD), assessed using the Personality Assessment Inventory-Borderline Scale (PAI-BOR).

## The present study

Our hypotheses, alongside our data collection and analysis strategies, were pre-registered (https://osf.io/5ek7b/). In this study, we detail the principal outcomes of this pre-registered study in relation to the revision of the ETMCQ. The aims of the analyses presented in this cross-sectional study were to: (a) develop a revised ETMCQ-R, with improved factor loadings; (b) examine the strength of associations between the updated scale and symptoms of mental health disorder, personality features, epistemic vice, psychological resilience, perceived social support, attachment style and history of childhood adversity; (c) explore the relationship between ETMCQ-R subscales and experimentally derived assessments of facial trustworthiness; and (d) explore the role of epistemic disruption as a mediator of the relationship between childhood adversity and current symptoms of mental health disorder.

## Method

### Procedure and participants

The revision of the ETMCQ involved a thorough theoretical review, analysis of the psychometric properties of the original scale along with its translations, and consultations with experts in the field. This process resulted in the addition of 9 new items, distributed evenly among the sub-factors of Mistrust, Trust and Credulity, thus expanding the scale to comprise 24 items. These additions aimed specifically at enhancing the construct validity of the ETMCQ, with special attention to improving the Trust scale.

The ETMCQ-R was completed in March 2023 by 525 individuals recruited through the survey platform Prolific (https://www.prolific.co), targeting a sample representative of the population of the UK. To ensure representativeness, the demographic distribution of participants was aligned with that of the UK in terms of age, gender and ethnicity. All participants were aged over 18, lived in the UK and were fluent in English. Demographic information was gathered, including age (grouped into 18–29, 30–39, 40–49, 50–59 and ≥60 years age brackets), annual household income (categorised as ‘below £30 000’, ‘£30 000 and above’ or ‘prefer not to answer’), educational attainment (options were secondary education, university degree, postgraduate degree, no formal qualifications or prefer not to answer), ethnicity (classified as ‘white’, ‘minoritised’ for smaller demographic groups or ‘prefer not to answer’), gender (options were ‘female’, ‘male’, ‘non-binary’ or ‘prefer not to answer’) and relationship status (‘married/in a relationship’, ‘single/widowed/divorced’ or ‘prefer not to answer’). All participants were required to provide written informed consent before taking part in this study. The authors assert that all procedures contributing to this work comply with the ethical standards of the relevant national and institutional committees on human experimentation and with the Helsinki Declaration of 1975, as revised in 2013. Ethical approval was granted by the University College London Research Ethics Committee (ethics number: 20129/001).

### Measures

The ETMCQ-R is based on the ETMCQ^1^ with the introduction of 9 new items, 3 for each sub-factor, creating a scale consisting of 24 items across three dimensions: Trust, Mistrust and Credulity. Participant responses were recorded using a 7-point Likert scale, with options ranging from ‘strongly disagree’ (= 1) to ‘strongly agree’ (= 7) and the midpoint being ‘neither agree nor disagree’ (= 4). High degrees of Trust, Mistrust and Credulity were identified by either strong agreement (= 7) or strong disagreement (= 1) with the statements provided.

The *Experiences in Close Relationships – Revised* (ECR–R)^31^ questionnaire is a 36-item self-assessment tool designed to gauge adult attachment styles, applying a 7-point Likert scale from ‘strongly disagree’ (= 1) to ‘strongly agree’. It consists of two subscales evaluating attachment anxiety and attachment avoidance separately. The ECR-R has excellent internal consistency (Cronbach’s *α* for avoidance = 0.95, for anxiety = 0.93) and reliability.^32^

The *Brief Symptom Inventory* (BSI)^33^ is a 53-item self-report instrument intended to assess symptoms of psychiatric disorder, using a 5-point Likert scale from ‘not at all’ (= 0) to ‘extremely’ (= 4). The BSI comprises nine subscales addressing various symptom dimensions: somatisation, obsession–compulsion, interpersonal sensitivity, depression, anxiety, hostility, phobic anxiety, paranoid ideation and psychoticism. It also features three global distress indices: the Global Severity Index (the aggregate of the nine subscales, plus four additional items not part of the subscales), the Positive Symptom Distress Index (the sum of non-zero response item values, divided by the total number of items receiving non-zero responses) and the Positive Symptom Total (the tally of all items with non-zero responses). The BSI has exceptional internal consistency (Cronbach’s *α* = 0.97), construct validity and reliability.

*The Personality Assessment Inventory-Borderline Scale* (PAI-BOR)^34^ is a 24-item self-report tool designed to identify primary characteristics of BPD, including emotional instability, identity issues, negative relationships and self-harm behaviours. This instrument uses a 4-point Likert scale ranging from ‘false’ (= 0) to ‘very true’ (= 3), with each subscale containing six items. The PAI-BOR has excellent internal consistency (Cronbach’s *α* = 0.84) and strong test–retest reliability.^34^

The *Brief Resilience Scale* (BRS)^35^ consists of six items that gauge an individual’s self-perceived ability to recover from stress. Responses are gathered using a 5-point Likert scale from ‘strongly disagree’ (= 1) to ‘strongly agree’ (= 5), incorporating both positively and negatively worded items. The BRS has robust internal consistency, with Cronbach’s *α* values ranging between 0.80 and 0.90, and reliable internal validity.

The *Epistemic Vice Scale* ^30^ is a 10-item self-report questionnaire designed to detect character traits that obstruct the acquisition, retention and sharing of knowledge. This recently developed scale includes two subscales, indifference and rigidity, each measuring different elements of epistemic vice.

The *Facial Trust Task* ^29^ measures initial reactions to facial trustworthiness. Participants are shown 70 adult facial images individually and are tasked with deciding whether each face seems trustworthy. This decision is made by pressing a ‘yes’ or ‘no’ button, capturing the participant’s instant judgment of each face’s trustworthiness. The task offers a direct way to assess participants’ immediate trust responses to facial features.

The *Multidimensional Scale of Perceived Social Support* (MSPSS)^36^ is a 12-item measure for evaluating perceived social support from family, friends and significant others. It features a 7-point Likert scale from ‘very strongly disagree’ (= 1) to ‘very strongly agree’ (= 7), with four items allocated to each subscale. The MSPSS has strong internal consistency (Cronbach’s *α* = 0.81) and adequate test–retest reliability.

The *Maltreatment and Abuse Chronology of Exposure* (MACE) scale^37^ is a 75-item questionnaire that assesses the intensity of ten types of childhood adversity: verbal abuse, non-verbal emotional abuse, physical abuse, sexual abuse, emotional neglect, physical neglect, peer emotional abuse, peer physical bullying, witnessing violence between parents and witnessing violence towards siblings. It uses a 10-point scale to quantify the variety of maltreatment experiences and also calculates a comprehensive score for the severity of childhood maltreatment up to the age of 18 (ranging from 0 to 100). The MACE has exceptional internal consistency (Cronbach’s *α* = 0.92) and overall reliability.

### Statistical analysis

The data analysis for this study was carried out using Stata (v.18.0 for Windows) and RStudio (v.4.4.1 for Windows).

#### Factor analyses

To uncover the underlying factor structure of the 24 items in the ETMCQ-R, exploratory factor analysis (EFA) was conducted. The threshold for identifying distinct factors was an eigenvalue exceeding 1.0, in line with Guttman’s^38^ recommendation. The items were treated as ordinal categorical variables, and cases with missing data were omitted from the analysis. Confirmatory factor analysis (CFA) was then used to confirm the three-factor structure noted in the original ETMCQ.^1^ During this phase, factor loadings were scrutinised, leading to the removal of the two items with the lowest loadings from each of the three factors to reduce overlap. Spearman correlations were used to examine the relationships between the factors of the ETMCQ-R. We deleted three items from the original ETMCQ: ‘I’d prefer to find things out for myself on the internet rather than asking people for information’ (Mistrust), ‘When I speak to different people, I find myself easily persuaded by what they say even if this is different from what I believed before’ (Credulity) and ‘In the past, I have misjudged who to believe and been taken advantage of’ (Credulity). In addition, we excluded three items from the 24-item revised version (two items for Trust and one for Mistrust). The full version of the scale is available from the corresponding author upon written request.

The model’s fit was assessed using several fit indices for both the unidimensional ETMCQ-R with all 24 items and the final, 18-item ETMCQ-R: the root mean square error of approximation (RMSEA), the comparative fit index (CFI), the Tucker–Lewis Index (TLI) and the standardised root mean square residual (SRMR). RMSEA and SRMR values below 0.08 were considered to indicate an acceptable fit, and values below 0.05 indicated a good fit. For the CFI and TLI, scores over 0.9 were acceptable, and values over 0.95 were seen as good, adhering to the standards set by Hu and Bentler^39^ and Schermelleh-Engel et al.^40^ An 18-item version was considered appropriate as having four to six items per factor is common practice, striking a balance between item and structure reliability and avoiding response fatigue.

#### Correlational analyses

Subsequently, the factors of the ETMCQ-R were correlated with a variety of developmental, psychopathological and psychological assessments to evaluate validity. Partial correlations were performed, adjusting for the demographic variables age, gender, annual income and educational attainment. A correction for multiple comparisons was enforced using a 5% false discovery rate (FDR), following the Benjamini–Hochberg FDR correction method.^41^ Attachment styles were defined through a median split of the anxious and avoidant subscales from the ECR-R questionnaire, with the medians set at 3.28 for anxious and 2.94 for avoidant. These classifications were then subjected to one-way ANOVA to investigate differences in epistemic stance among the categories.

#### Mediation analyses

Lastly, the potential mediating effect of individual ETMCQ-R factors on the link between childhood adversity (evaluated by the MACE) and psychopathology (assessed by the BSI and PAI-BOR) was explored through mediation analysis using the PROCESS macro in R.^42^ This analysis aimed to replicate findings from the earlier study with the original ETMCQ.^1^ The analysis calculated the indirect impact of adversity through each ETMCQ-R factor in two models, one for each measure of psychopathology, while adjusting for demographic factors. Bias-corrected bootstrapped confidence intervals for the indirect effects were derived using 5000 bootstrap samples.

## Results

### Demographic data

The analysis included a total of 525 adults. Within this group, the largest age category was 18–29 years, comprising 22.5% of the sample (*n* = 118), whereas the smallest was the 40–49 age group, making up 16.8% (*n* = 88). A significant portion of the participants (*n* = 298; 56.8%) reported an annual household income of £30 000 or more. In terms of educational attainment, 231 participants (44.0%) had obtained a university degree. The sample was predominantly white (*n* = 442; 84.2%) and a slight majority was female (*n* = 272; 51.8%). The majority of respondents (340; 64.8%) were either married or in a relationship. The demographic composition of the sample reflected the UK population’s distribution in terms of age, sex and ethnicity (see Table 1).

**Table 1:** Demographic data

Category		*n* (%)
	Full sample	525
Age band	18–29	118 (22.5)
	30–39	102 (19.4)
	40–49	88 (16.8)
	50–59	105 (20.0)
	≥60	112 (21.3)
Annual household income	Low: <£30 000	197 (37.5)
	High: ≥£30 000	298 (56.8)
	Prefer not to answer	30 (5.7)
Educational level	Secondary	190 (36.2)
	University degree	231 (44.0)
	Postgraduate	100 (19.1)
	Prefer not to answer	3 (0.6)
	No formal qualifications	1 (0.2)
Ethnicity	White	442 (84.2)
	Minoritised ethnicity/other	80 (15.2)
	Prefer not to answer	3 (0.6)
Gender	Female	272 (51.8)
	Male	244 (46.5)
	Non-binary	5 (1.0)
	Prefer not to answer	4 (0.8)
Relationship status	Married/in relationship	340 (64.8)
	Single/widowed/divorced	182 (34.7)
	Prefer not to answer	3 (0.6)

### ETMCQ-R factor structure

In accordance with the first ETMCQ study,^1^ EFA confirmed that a three-factor solution best matched the data, based on the eigenvalue guideline. The eigenvalues for the factors were: 5.87 for the first factor, which accounted for 16.1% of the total variance; 3.22 for the second factor, contributing to 15.0% of the variance; and 1.28 for the third factor, explaining 12.1% of the variance. All additional eigenvalues were below the threshold of 1 and therefore did not meet the established level for identifying a distinct factor (the next highest was 0.83).

Employing an oblique (promax) rotation for principal factor extraction, the factor analysis identified that each of the three factors – (a) Trust; (b) Mistrust; (b) Credulity – comprised eight items with loadings above 0.25. The distribution of items across these factors generally aligned with expectations (see Table 2). However, there was an anomaly with item 16 (‘I frequently feel that I don’t know what to think about what someone is telling me, even when I understand what they said’), which unexpectedly associated with the Mistrust factor rather than with Credulity, exhibiting factor loadings of 0.46 for Mistrust and 0.45 for Credulity. This situation prompted consideration of whether item 16 should be retained or removed, based on its comparative loadings and the conceptual alignment with the respective factors.

**Table 2:** Factor loading results from the exploratory factor analysis

Item	Credulity	Mistrust	Trust
Item 1	−0.22	0.23	**−0.54**
Item 2	0.13	−0.04	**−0.71**
Item 3	0.27	**−0.51**	0.10
Item 4	0.05	**0.63**	0.03
Item 5	**−0.79**	0.03	0.08
Item 6	**0.77**	−0.08	−0.04
Item 7	0.03	0.10	**0.67**
Item 8	−0.23	0.17	**0.75**
Item 9	−0.06	**0.75**	0.14
Item 10	0.04	**0.67**	0.09
Item 11	**0.63**	0.18	−0.08
Item 12	**−0.91**	0.06	0.05
Item 13	0.12	−0.14	**0.61**
Item 14	0.10	**0.40**	−0.14
Item 15	**−0.62**	−0.30	−0.01
Item 16	0.45	**0.46**	0.02
Item 17	0.01	0.05	**−0.51**
Item 18	0.10	−0.13	**−0.51**
Item 19	−0.05	**−0.60**	−0.04
Item 20	**0.39**	−0.04	0.31
Item 21	0.26	**0.49**	−0.04
Item 22	**0.44**	0.33	−0.01
Item 23	0.02	−0.02	**0.27**
Item 24	−0.11	**−0.61**	0.01

Bold indicates the highest factor loading result per item.

CFA was carried out on the sample to assess the fit of various potential models. These models included an unconstrained (unidimensional) model, a 24-item three-factor model and an 18-item three-factor model; further details are provided in the study’s pre-registration. The 18-item model emerged by excluding the two items with the weakest factor loadings from each dimension. The unconstrained model showed acceptable fit indicators (CFI = 0.935; TLI = 0.928; SRMR = 0.086; RMSEA = 0.105; 95% CI [0.100, 0.110]), while the 18-item model displayed improved fit indices (CFI = 0.971; TLI = 0.966; SRMR = 0.067; RMSEA = 0.083; 95% CI [0.076, 0.090]). Thus the 18-item, three-factor model was retained as the final ETMCQ-R on the basis of having the best fit.

Significant relationships were noted between the factors. Trust and Mistrust exhibited a negative correlation (*r* = −0.24, *p* < 0.001), and Mistrust and Credulity had a positive correlation (*r* = 0.59, *p* < 0.001). No significant relationship was identified between Trust and Credulity.

Table 3 compares the fit statistics of the original ETMCQ with those of the ETMCQ-R (i.e. using both versions of the questionnaire with the new sample), highlighting the enhancements and overall improvement in model fit following the revision. These comparisons demonstrate that the ETMCQ-R possesses greater model fit compared with its predecessor.

**Table 3:** Model fit for the original Epistemic Trust, Mistrust and Credulity Questionnaire (ETMCQ) and the Revised ETMCQ (ETMCQ-R)

	Original ETMCQ				ETMCQ-R			
Model	CFI	TLI	SRMR	RMSEA (95% CI)	CFI	TLI	SRMR	RMSEA (95% CI)
Unidimensional	0.394	0.313	0.136	0.209 (0.2–0.22)	0.761	0.739	0.144	0.199 (0.195–0.204)
3-factor, 15 items	0.849	0.825	0.075	0.105 (0.1–0.12)	0.935	0.928	0.086	0.105 (0.100–0.110)
3-factor, 18 items	0.880	0.855	0.069	0.111 (0.10–0.12)	0.971	0.966	0.067	0.083 (0.076–0.090)

CFI, Comparative Fit Index; TLI, Tucker–Lewis Index; SRMR, standardised root mean square residual; RMSEA, root mean square error of approximation.

### Correlation analyses

The Spearman correlation coefficients between the ETMCQ-R subscales and related developmental, psychological and psychopathology measures were examined while controlling for demographic features (age, gender, annual income and level of education) (Table 4).

**Table 4:** Correlation analyses

Measure		Trust	Mistrust	Credulity
Psychopathology	PAI-BOR: Affective instability	−0.13^*^	0.50^**^	0.38^**^
	PAI-BOR: Identity problems	−0.03	0.47^**^	0.46^**^
	PAI-BOR: Negative relationships	−0.13^*^	0.43^**^	0.29^**^
	PAI-BOR: Self-harm	−0.04	0.31^**^	0.40^**^
	PAI-BOR: Total score	−0.10^*^	0.54^**^	0.48^**^
	BSI: Total score	−0.12^*^	0.41^**^	0.36^**^
Attachment	ECR-R: Anxious	−0.11^*^	0.43^**^	0.44^**^
	ECR-R: Avoidance	−0.40^**^	0.34^**^	0.29^**^
Social support	MSPSS: Significant other	0.35^**^	−0.17^**^	−0.19^**^
	MSPSS: Family	0.40^**^	−0.25^**^	−0.20^**^
	MSPSS: Friends	0.44^**^	−0.32^**^	−0.20^**^
	MSPSS: Total	0.46^**^	−0.30^**^	−0.22^**^
Childhood adversity	MACE: Multiplicity	−0.11^*^	0.29^**^	0.18^**^
	MACE: Severity	−0.10^*^	0.29^**^	0.20^**^
Resilience	BRS Total	0.09	−0.31^**^	−0.36^**^
Epistemic vice	Rigidity	−0.04	0.22^**^	0.16^**^
	Indifference	−0.22^**^	0.22^**^	0.27^**^
	EV mean	−0.13^*^	0.27^**^	0.25^**^
Facial trust measure		0.11^*^	−0.18^**^	−0.02

Spearman correlation coefficients between Revised Epistemic Trust, Mistrust and Credulity Questionnaire (ETMCQ-R) subscales and related developmental, psychological and psychopathology measures, controlling for demographic variables (age, gender, annual income and level of education). PAI-BOR, Personality Assessment Inventory-Borderline Scale; BSI, Brief Symptom Inventory; ECR-R, Experiences in Close Relationships – Revised; MSPSS, Multidimensional Scale of Perceived Social Support; MACE, Maltreatment and Abuse Chronology of Exposure; BRS, Brief Resilience Scale; EV, epistemic vice.

^*^p < 0.05, ^**^ *p* < 0.001.

Significant negative correlations were identified between Trust (*r* = −0.12) and higher scores on the global psychopathology severity index as assessed by the BSI. Conversely, Mistrust (*r* = 0.41) and Credulity (*r* = 0.36) showed positive correlations with higher scores on the same index, which were slightly more pronounced than those reported in the previous study (*r* = −0.10 for Trust, *r* = 0.40 for Mistrust and *r* = 0.33 for Credulity).^1^ Regarding the assessment of psychopathology through the measurement of borderline features, our findings were somewhat stronger than the results found using the global psychopathology severity index: Trust negatively correlated with higher PAI-BOR scores (*r* = −0.10), whereas Mistrust and Credulity were positively correlated with PAI-BOR scores (*r* = 0.54 and *r* = 0.48, respectively).

Concerning the other two domains of functioning, resilience and perceived social support, we observed that resilience had a negative association with both Mistrust (*r* = −0.31) and Credulity (*r* = −0.36); there was no significant correlation with Trust (*r* = 0.09) (see Table 5). However, the Trust subscale was significantly associated with perceived social support (*r* = 0.46), showing a stronger correlation than both Mistrust and Credulity, which were negatively correlated with perceived social support (*r* = −0.30 and *r* = −0.22, respectively).

**Table 5:** Correlations between the Revised ETMCQ (ETMCQ-R) and resilience and perceived social support

Subscale	1	2	3	4	5	6	7	8
1. ETMCQ-R (Trust)	1							
2. ETMCQ-R (Mistrust)	−0.24^**^	1						
3. ETMCQ-R (Credulity)	−0.1^*^	0.58^**^	1					
4. BRS (Total)	0.09	−0.31^**^	−0.36^**^	1				
5. MSPSS (Family)	0.39^**^	−0.25^**^	−0.2^**^	0.15^**^	1			
6. MSPSS (Friends)	0.44^**^	−0.32^**^	−0.22^**^	0.21^**^	0.48^**^	1		
7. MSPSS (Significant other)	0.34^**^	−0.19^**^	−0.2^**^	0.1^*^	0.59^**^	0.41^**^	1	
8. MSPSS (Total)	0.45^**^	−0.31^**^	−0.23^**^	0.19^**^	0.85^**^	0.76^**^	0.78^**^	1

BRS, Brief Resilience Scale; MSPSS, Multidimensional Scale of Perceived Social Support.

**Fig. 1:**
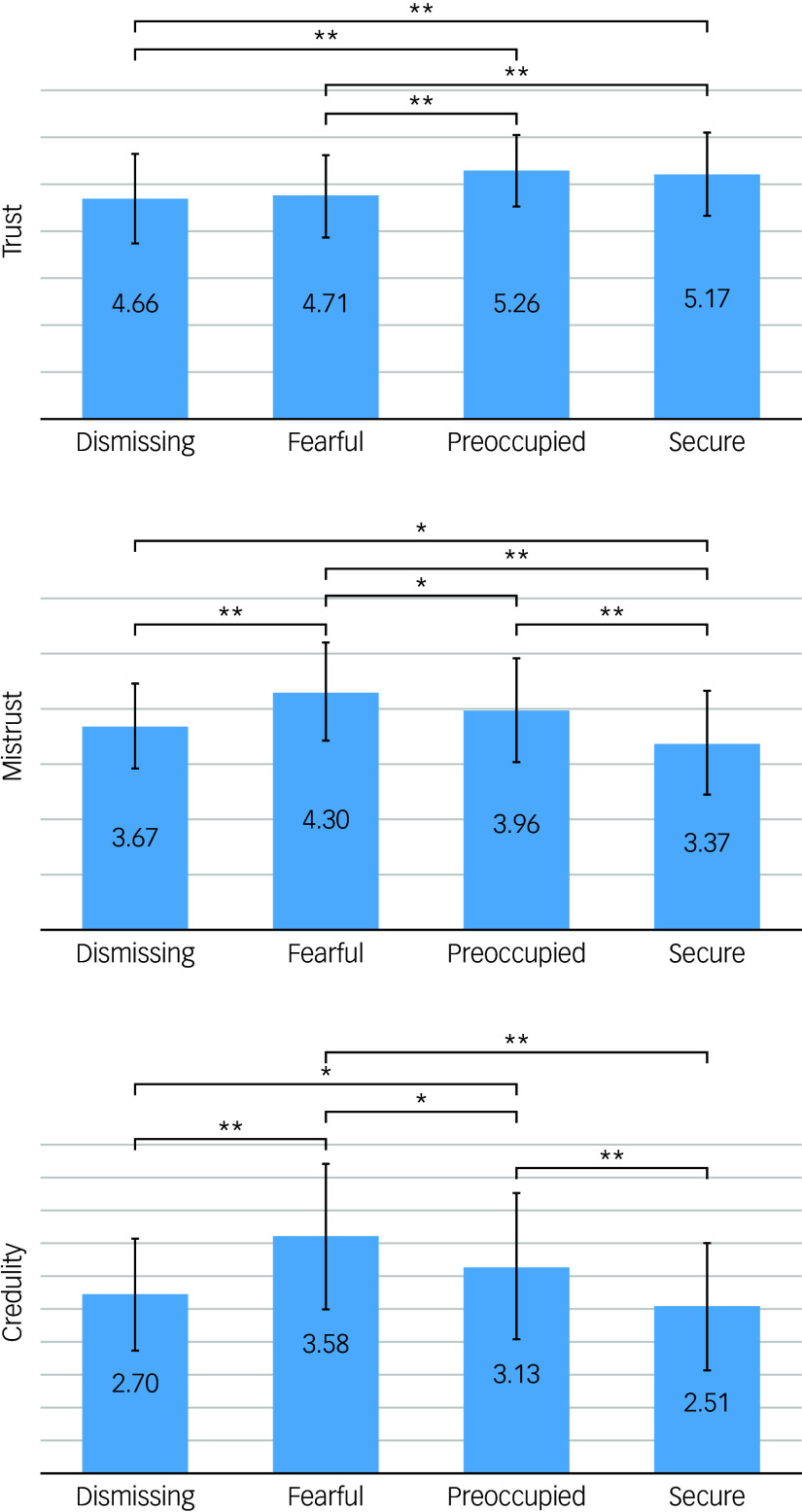
Comparisons of individual epistemic stance subscale scores between attachment groups. Assessed using the Experiences in Close Relationships Scale – Revised (ECR–R). Group comparisons tested using pairwise *t*-tests. ^*^ *p* < 0.05, ^**^ *p* < 0.001.

The study also examined the relationship between different attachment styles and epistemic stance, using a methodology akin to the one employed in the original ETMCQ study.^1^ Attachment styles were classified using a median split of the anxious and avoidant subscale scores, categorising 199 participants as ‘secure’, 83 as ‘preoccupied’, 67 as ‘dismissing’ and 176 as ‘fearful’.^31^ Significant differences between the attachment style groups were observed in the Trust subscale (*F*(3,511) = 14.17, *p* < 0.001) (see Fig. 1). The Secure and Preoccupied groups scored significantly higher on Trust compared with the Dismissing and Fearful groups. There was no significant difference in Trust scores between the Secure and Preoccupied groups, nor between the Dismissing and Fearful groups. This echoed our findings regarding the Trust subscale from the initial ETMCQ, where comparisons between groups demonstrated that the Secure and Preoccupied groups scored significantly higher than the Dismissing and Fearful groups, without significant differences in Secure versus Preoccupied or Dismissing versus Fearful comparisons.

For the Mistrust subscale, significant differences were found between the groups (*F*(3,511) = 33, *p* < 0.001). The Secure group exhibited significantly lower mean scores on Mistrust compared with all insecure groups. The Fearful group had the highest mean scores on Mistrust, significantly exceeding those of all other groups. There was no significant difference in Mistrust scores between the Dismissing and Preoccupied groups. These results mirrored those from our previous study, which similarly reported that the Secure group’s mean Mistrust score was significantly lower than that of the other three groups, and the Fearful group’s mean score was notably higher than the others. For the Credulity subscale, significant differences were noted once more (*F*(3,511) = 35.52, *p* < 0.001). The Fearful and Preoccupied groups scored significantly higher on Credulity compared with the other groups. The Dismissing group’s Credulity scores were not significantly different from those of the Secure group, reaffirming the outcomes from our prior study (see Fig. 1).

Consistent with our expectations, both the diversity (the number of different types) and the total instances (the total number of occasions) of maltreatment demonstrated a negative association with Trust (*r* = −0.11 for diversity and *r* = −0.10 for total instances), whereas both metrics showed positive associations with Mistrust (0.29 for diversity and 0.29 for total instances) and Credulity (0.18 for diversity and 0.20 for total instances). When examining the subscales that measure different types of adverse experiences, the most pronounced effects were observed in the correlations between parental verbal abuse, sexual abuse and neglect with Mistrust. Regarding Credulity, the strongest associations were with sexual abuse and parental verbal abuse, followed by peer verbal and physical abuse, as detailed in Table 6. A further exploratory analysis, which was not pre-registered, examined the correlation between trust and BPD scoring among participants in the top 20% on the MACE scale; it was found that within this group, the Trust factor was more negatively correlated with BPD than in the bottom 80% (see Supplementary Table 1 available at https://doi.org/10.1192/bjo.2025.10813).

**Table 6:** Correlation analysis between the subscales of the Revised Epistemic Trust, Mistrust and Credulity Questionnaire (ETMCQ-R) and the Maltreatment and Abuse Chronology of Exposure (MACE) subscales of adverse childhood experiences

Subscale	1	2	3	4	5	6	7	8	9	10	11	12	13
1. ETMCQ-R (Trust)	–												
2. ETMCQ-R (Mistrust)	−0.24^**^	–											
3. ETMCQ-R (Credulity)	−0.08	0.55^**^	–										
4. MACE (Sexual abuse)	0.02	0.21^**^	0.18^**^	–									
5. MACE (Parental verbal abuse)	−0.03	0.24^**^	0.18^**^	0.26^**^	–								
6. MACE (Non-verbal emotional abuse)	−0.02	0.2^**^	0.12^*^	0.23^**^	0.62^**^	–							
7. MACE (Parental physical maltreatment)	−0.1^*^	0.18^**^	0.1^*^	0.15^*^	0.52^**^	0.42^**^	–						
8. MACE (interpersonal violence)	−0.01	0.06	0.05	0.11^*^	0.43^**^	0.35^**^	0.32^**^	–					
9. MACE (Witnessing violence to sibling)	0.01	0.12^*^	0.09	0.11^*^	0.29^**^	0.24^**^	0.37^**^	0.19^**^	–				
10. MACE (Peer verbal abuse)	−0.06	0.19^**^	0.17^**^	0.22^**^	0.5^**^	0.38^**^	0.35^**^	0.27^**^	0.22^**^	–			
11. MACE (Peer physical bullying)	−0.05	0.2^**^	0.17^**^	0.14^*^	0.36^**^	0.29^**^	0.37^**^	0.18^**^	0.22^**^	0.68^**^	–		
12. MACE (Emotional neglect)	−0.13^*^	0.21^**^	0.13^*^	0.22^**^	0.48^**^	0.57^**^	0.27^**^	0.3^**^	0.16^**^	0.28^**^	0.19^**^	–	
13. MACE (Physical neglect)	−0.1^*^	0.16^*^	0.13^*^	0.16^*^	0.27^**^	0.39^**^	0.19^**^	0.21^**^	0.09	0.21^**^	0.18^**^	0.49^**^	–

Spearman correlation coefficients between ETMCQ-R subscales and the MACE subscales of adverse childhood experiences controlling for demographic variables (age, gender, annual income and level of education).

^*^p < 0.05 and ^**^p < 0.001.

The final set of correlation analyses focused on the Epistemic Vice Scale and participants’ behaviour in the facial trust task (see Table 4). The results for the Epistemic Vice Scale indicated moderate correlations between epistemic disruption and epistemic vice: Credulity was more strongly correlated with epistemic indifference (0.27) than with epistemic rigidity (0.16), while Mistrust displayed equal correlations with both epistemic vice factors (0.22 for indifference and 0.22 for rigidity). The outcomes from the Todorov facial trust task were surprisingly weak: Trust showed a slight positive correlation with the propensity to perceive faces as trustworthy (0.11) and Credulity showed a minor negative correlation (−0.02). Mistrust presented a somewhat stronger negative correlation (−0.18).

### Relationship between childhood adversity, epistemic stance and psychopathology

Two distinct pre-registered mediation analyses were performed to explore the link between childhood adversity, as quantified by the MACE, and psychopathology, gauged through two different instruments: the BSI for the first model and the PAI-BOR for the second. In both models, factors such as age, gender, income and educational attainment were accounted for as covariates to mitigate their possible effects.

**Fig. 2:**
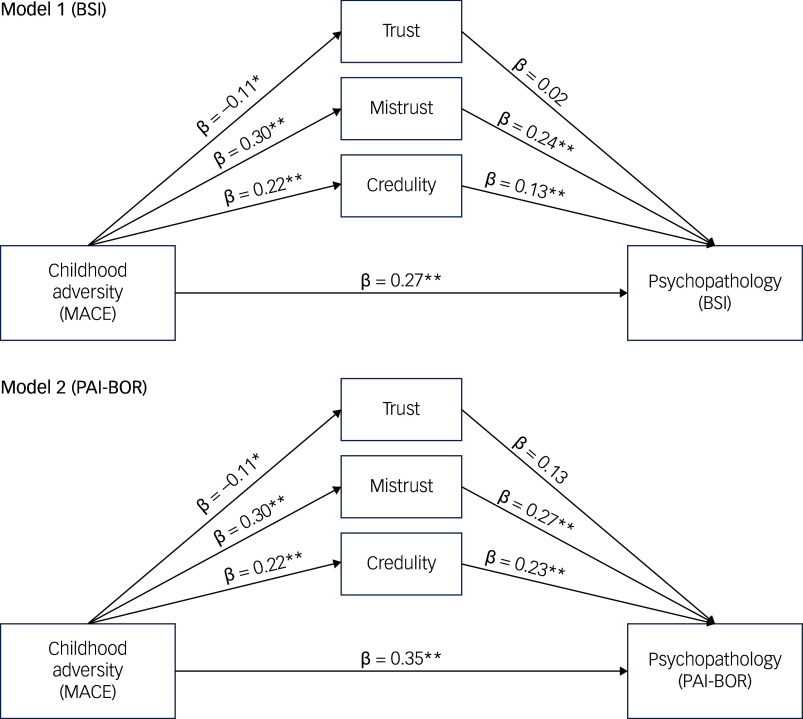
Schematic of the mediation model of the three Revised Epistemic Trust, Mistrust and Credulity Questionnaire (ETMCQ-R) dimensions between childhood adversity (Maltreatment and Abuse Chronology of Exposure (MACE)) and psychopathology (Model 1: Brief Symptom Inventory (BSI); Model 2: Personality Assessment Inventory-Borderline Scale (PAI-BOR)). See Supplementary Text 1 for a narrative description of the paths depicted in Fig. 2.

The analysis revealed a significant direct effect of ACEs on general psychological distress (β = 0.274, s.e. = 0.044, *p* < 0.001, 95% CI [0.188, 0.360]), indicating that individuals with higher levels of ACEs reported greater distress (see Fig. 2; see also Supplementary Text 1 for a narrative description of the paths depicted in Fig. 2). In addition to this direct effect, ACEs also influenced distress indirectly through epistemic stance. Specifically, the indirect effect of ACEs on distress via mistrust was significant (β = 0.072, s.e. = 0.018, *p* < 0.001, 95% CI [0.037, 0.108]), and mistrust in turn mediated higher levels of distress. A smaller but still significant indirect effect was observed through credulity (β = 0.028, s.e. = 0.014, *p* = 0.038, 95% CI [0.002, 0.055]), indicating a similar mediating role. However, the indirect effect via trust was not statistically significant (β = −0.002, s.e. = 0.005, *p* = 0.729, 95% CI [−0.011, 0.008]). These findings support the interpretation that ACEs contribute to psychological distress not only directly but also indirectly through increased mistrust and credulity.

The analysis also demonstrated a significant direct effect of ACEs on borderline personality features as measured by the PAI-BOR total score (β = 0.351, s.e. = 0.034, *p* < 0.001, 95% CI [0.284, 0.418]), indicating that greater exposure to childhood adversity was associated with elevated borderline traits. Beyond this direct effect, the indirect effect through mistrust was significant (β = 0.083, s.e. = 0.018, *p* < 0.001, 95% CI [0.048, 0.118]), suggesting that higher levels of childhood adversity predicted increased mistrust, which in turn was associated with more pronounced borderline features. A smaller but also significant indirect effect was observed through credulity (β = 0.050, s.e. = 0.014, *p* < 0.001, 95% CI [0.023, 0.078]), supporting a role for overly trusting interpersonal schemas in this pathway. In contrast, the indirect effect via trust was not statistically significant (β = −0.001, s.e. = 0.004, *p* = 0.720, 95% CI [−0.010, 0.007]). These results indicate that the impact of ACEs on borderline psychopathology is partly mediated by epistemic mistrust and credulity, whereas positive expectations of trust were not found to play a significant mediating role.

## Discussion

In this study, we assessed and validated an updated version of the Epistemic Trust, Mistrust and Credulity Questionnaire (the ETMCQ-R), concentrating on its structure and psychometric qualities. We undertook the revision of the scale to attempt to improve the performance of the Trust subscale, the factor loading and the reliability of certain items. The findings confirmed that the questionnaire’s three-factor model, consisting of Trust, Mistrust and Credulity, maintained its efficacy. The Trust subscale was found to perform better in relation to correlational findings, and demonstrated a superior fit with the data compared with the original ETMCQ. Furthermore, the scale’s consistency was improved in comparison with the previous version.

Additionally, this research contributed further evidence supporting the concurrent validity of the ETMCQ-R. We have predicted and found strong associations between mental disorder in the form of general psychopathology^33^ and epistemic disruption. In keeping with our theoretical model, results displayed a consistent pattern of correlations with general symptoms of mental health disorder as measured by the BSI. This consistency underscores a link between these facets of epistemic stance and general psychopathology. An important expansion of this study was the inclusion of a measure for borderline features, the PAI-BOR,^34^ prompted by the assumption that epistemic disruption could be particularly significant in the context of borderline pathology. Indeed, the correlations detected between the PAI-BOR total scores and elements of epistemic stance were more pronounced than those with the BSI, indicating a potentially greater influence of epistemic stance on borderline pathology than on more general symptoms of mental disorder. Thus, our findings on the relationship between borderline features and epistemic disruption provide a valuable proof of concept relevant to the clinical implications of such disruption.

In relation to these findings, we can explore how and why we have proposed the construct of epistemic trust to be central to our model of developmental psychopathology. Our earlier work on attachment, mentalising and developmental psychopathology tended to focus almost exclusively on the relational processes between the primary caregiver and the young child in the early years of life.^43^ Our more recent emphasis on the role of social communication and epistemic trust has led us to create a developmental framework that additionally accommodates the impact of the wider environment and a wider range of social-cognitive interactions.^2^ Thus, the impact of a non-mentalising social environment around the caregiver–child dyad is registered in the mentalising profile of the developing child. In addition, the impact of a non-mentalising social system is felt in terms of openness to social learning, generating closure (i.e. entrenched epistemic mistrust) or a compromised ability to exercise due epistemic vigilance (i.e. epistemic credulity). The concept of the internal working model (IWM) is well established in attachment theory.^44^ We suggest that processes at work here could be analogously understood as an internal working model of the community (IWM-C): that is, a way of thinking about the safety and reliability of social communication not just in the context of immediate attachment relationships but also in relation to alloparenting figures and environments such as schools. Just as attachment processes shape the IWM, epistemic stance shapes the IWM-C. Adaptive and proportionate epistemic trust can provide access to other minds that allows individuals to learn from and rethink how they might navigate a shared social environment; closure of this access undermines resilience and cognitive flexibility, and breakdowns in epistemic trust might explain the striking and strong research finding linking a lack of social support with increased risk of mental ill health.^45^

The outcomes from the Epistemic Vice Scale and the facial trust assessment offer intriguing insights into what might be specific about the socially nested epistemic trust that we have described in our model. For the Epistemic Vice Scale, Trust showed negative correlations with both rigidity and indifference, whereas Mistrust and Credulity showed positive associations. However, the strength of these correlations indicates that, although related, the Epistemic Vice Scale and the ETMCQ-R assess somewhat distinct social-cognitive processes. Importantly, epistemic stance had larger associations with psychopathology, encompassing both general symptoms and borderline features, than epistemic vice. This underscores the impact of challenges in assimilating new information from others, as opposed to broader issues in updating knowledge, on functioning. This impediment restricts opportunities for learning from diverse viewpoints and the adaptive benefits of social interactions^2^ and has been shown to be linked to a propensity for distorted social learning in the context of conspiracy beliefs.^16,46^

In the facial trust task, correlations were lower than anticipated, reflecting the distinction between the domains of interpersonal trust evaluated in this task and epistemic trust. Epistemic mistrust exhibited a minor negative correlation with perceptions of facial trustworthiness, whereas the correlations for Trust and Credulity were negligible and not significant. It is recognised that connections between self-reported measures and experimental assessments of the same concept are often small to modest.^47^ We interpret this result as a valuable possible step in showing the differentiation between epistemic trust, as we have it described in our model, and general trust. These results also hint at a more nuanced mechanism involving epistemic attitudes towards social communication and its link to psychopathology, especially symptoms related to borderline personality. The findings align with recent research indicating that individuals diagnosed with BPD rate faces as being trustworthy similarly to control groups, but show heightened sensitivity to untrustworthy faces.^48^

Regarding resilience, the outcomes paralleled those observed in psychopathology, with Trust displaying a positive but modest correlation with resilience, and both Mistrust and Credulity exhibiting stronger negative correlations. Trust was more closely associated with perceived social support, highlighting its potential for enhancing resilience through social bonds. These findings corroborate our hypotheses and the earlier conceptualisation of epistemic stance as a marker of openness to social learning, thereby facilitating adaptation and predominantly supporting health maintenance (also known as salutogenesis).^2^

In analysing the connections between epistemic trust factors and attachment styles, our observations closely matched those of the initial validation study of the ETMCQ. The trust factor demonstrated a strong correlation with preoccupied and secure attachment styles, while showing weaker associations with dismissive and fearful attachment styles. This finding is in line with attachment theory, which posits that individuals with secure or preoccupied attachment styles are likely to adopt a more trusting stance towards sources of information and knowledge.^49^ Notably, although the mistrust factor was, as expected, most strongly linked to a fearful attachment style, it was slightly more associated with a preoccupied attachment style than with a dismissing one, contrary to initial predictions and differing from the findings of the original study. Despite this unexpected result, it effectively differentiated between secure and insecure groups and retained the lowest correlation with the secure attachment style, mirroring the results of the initial study. In agreement with the previous study, the credulity factor was predominantly connected with fearful and preoccupied attachment styles, and less so with secure and dismissive styles.

Both the diversity and total incidents of exposure to childhood adversity were positively correlated with mistrust and credulity. This finding aligns with the understanding that maltreatment may lead to later challenges in adaptation in adulthood by affecting social learning through scepticism and an inability to accurately discern reliable, and thus potentially helpful, sources of information. The mediation analyses underscored this: both epistemic mistrust and credulity were found to mediate the relationship between childhood adversity and psychopathology, with stronger mediation relationships found for borderline features than for general psychopathology. This result provides valuable evidence for the developmental framework we have suggested – namely, that childhood adversity can disrupt the capacity for social learning both within and beyond attachment relationships – the IWM-C – which in turn increases risk for psychopathology. In an exploratory analysis, not pre-registered, examining the correlation between trust and BPD scoring among participants in the top 20% on the MACE scale, it was found that the Trust factor was more negatively correlated with BPD and trauma within this group than in the bottom 80% (see Supplementary Table 1). This suggests that the generally weak correlations for trust that were observed may be due to the studies being conducted in community, rather than clinical, samples. It highlights the importance of conducting further research in clinical groups to understand how trust might influence the development of psychopathology, and how facets of epistemic stance differentially predict response to treatment.^27^

There are several limitations of this study that must be noted. First, the sample population was community-based rather than a clinical group, despite our research questions pertaining to risk for psychopathology. In particular, given our interest in the relationship between BPD and epistemic trust, further research exploring epistemic trust in a sample of individuals diagnosed with BPD is needed. Second, our study is concerned with domains related to developmental psychopathology – in particular, attachment patterns and exposure to childhood adversity – but is cross-sectional rather than longitudinal in design. Given the developmental sequence, we consider it reasonable to suggest that these domains impact on current functioning, but we cannot state this with any certainty until results from longitudinal studies are available in order to explore the causal relationships between the variables of interest. Third, our study relied on self-report measures as well as a retrospective assessment of childhood adversity. Our study began a process of linking self-report measures with experimental designs, via the use of the facial trust measure devised by Todorov et al,^29^ but further experimental tasks that assess social learning are necessary to test whether the ETMCQ-R is correlated to individual differences in behaviour.

In conclusion, the ETMCQ-R was found to be a valid and reliable measure to assess epistemic stance, with improved factor loadings compared with the original version of the ETMCQ. In addition, correlations between the updated scale and symptoms of mental health disorder, personality features, psychological resilience, perceived social support, attachment style and history of childhood adversity were found, which replicate and strengthen findings obtained with the original ETMCQ. Correlations between the ETMCQ-R and both the Epistemic Vice Scale and assessments of facial trustworthiness indicate that epistemic trust, conceptualised as openness to the social communication of information, does appear to be a distinct (although overlapping) construct from both general trust and epistemic trust conceptualised as an abstract process. Finally, and important for our developmental framework, epistemic mistrust and credulity were found to mediate the relationship between exposure to childhood adversity and mental health outcomes, with stronger findings emerging in relation to borderline features as an outcome. In the context of predicting psychotherapeutic outcome, the more robust and reliable updated version of the ETMCQ presented here may provide a more accurate measure of the social-communicative capacities of an individual receiving psychotherapy, which could influence treatment response and help explain the relationship between therapeutic alliance and treatment outcome.

## Supporting information

Campbell et al. supplementary material Campbell et al. supplementary material

## Data Availability

The data that support the findings of this study are available from the corresponding author, C.C., upon reasonable request.

## Supplementary material

The supplementary material is available online at https://doi.org/10.1192/bjo.2025.10813
