# Supplementary material for: Development and validation of the Revised Epistemic Trust, Mistrust and Credulity Questionnaire (ETMCQ-R)
Source: BJPsych Open. 2025 Sep 1;11(5):e191. doi: 10.1192/bjo.2025.10813 (PMC12451556; doi:10.1192/bjo.2025.10813)
Supplement: Campbell et al. supplementary material [file S2056472425108132sup001.docx]

**Correlation analyses: Table 7 (Divided by 20/80 for those scoring on the MACE: Severity)**

|  |  | **Top 20% for MACE (Severity)** | | | **Bottom 80% for MACE (Severity)** | | |
| --- | --- | --- | --- | --- | --- | --- | --- |
|  |  | **Trust** | **Mistrust** | **Credulity** | **Trust** | **Mistrust** | **Credulity** |
| **Psychopathology** | PAI-BOR: Affective instability | -0.19 | 0.53** | 0.28* | -0.1* | 0.43** | 0.38** |
|  | PAI-BOR: Identity problems | -0.15 | 0.5** | 0.45** | 0.02 | 0.4** | 0.44** |
|  | PAI-BOR: Negative relationships | -0.16 | 0.41** | 0.37** | -0.11* | 0.37** | 0.25** |
|  | PAI-BOR: Self-harm | -0.3* | 0.27* | 0.24* | 0.04 | 0.28** | 0.42** |
|  | PAI-BOR: Total score | -0.27* | 0.54** | 0.41** | -0.05 | 0.48** | 0.48** |
|  | BSI: Total score | -0.13 | 0.29* | 0.22* | -0.1 | 0.38** | 0.36** |
| **Attachment** | ECR-R: Anxious | -0.07 | 0.46** | 0.5** | -0.1* | 0.4** | 0.41** |
|  | ECR-R: Avoidance | -0.42** | 0.23* | 0.22* | -0.41** | 0.35** | 0.29** |
| **Social** **Support** | MSPSS: Significant other | 0.23* | -0.11 | -0.21* | 0.37** | -0.18** | -0.17* |
|  | MSPSS: Family | 0.4** | -0.26* | -0.14 | 0.39** | -0.21** | -0.16* |
|  | MSPSS: Friends | 0.41** | -0.35** | -0.31* | 0.45** | -0.28** | -0.16* |
|  | MSPSS: Total | 0.44** | -0.33* | -0.26* | 0.46** | -0.27** | -0.17** |
| **Childhood Adversity** | MACE: Multiplicity | -0.27* | 0.3* | 0.11 | -0.09 | 0.2** | 0.14* |
|  | MACE: Severity | -0.29* | 0.29* | 0.16 | -0.08 | 0.21** | 0.17* |
| **Resilience** | BRS Total | 0.07 | -0.22* | -0.26* | 0.07 | -0.32** | -0.34** |
| **Epistemic Vice** | Rigidity | 0.03 | 0.18 | -0.03 | -0.05 | 0.24** | 0.2** |
|  | Indifference | -0.15 | 0.09 | 0 | -0.22** | 0.3** | 0.34** |
|  | EV mean | -0.03 | 0.16 | -0.03 | -0.14* | 0.32** | 0.31** |
| **Facial Trust measure** |  | 0.2* | -0.17 | 0.13 | 0.11* | -0.16* | -0.01 |

**Supplementary Text 1 describing the paths in Figure 2**

In the model predicting general psychological distress (BSI) – see Figure 2 – adverse childhood experiences (ACEs) were significantly associated with lower levels of trust, β = –0.114, SE = 0.046, p = .014, 95% CI [–0.205, –0.023], higher levels of mistrust, β = 0.303, SE = 0.043, p < .001, 95% CI [0.219, 0.386], and greater credulity, β = 0.215, SE = 0.044, p < .001, 95% CI [0.129, 0.300]. Of the three interpersonal schemas, only mistrust and credulity were significant predictors of distress. Mistrust was positively associated with distress, β = 0.239, SE = 0.052, p < .001, 95% CI [0.137, 0.341], and credulity also showed a significant association, β = 0.132, SE = 0.057, p = .022, 95% CI [0.019, 0.244]. In contrast, trust did not significantly predict distress, β = 0.015, SE = 0.042, p = .724, 95% CI [–0.067, 0.096]. These findings indicate that while ACEs influence epistemic stance, only mistrust and credulity appear to contribute meaningfully to psychological distress.

In the model using borderline personality features (PAI-BOR) as the outcome – see Figure 2 – ACEs were again significantly associated with lower levels of trust, β = –0.114, SE = 0.046, p = .014, 95% CI [–0.205, –0.023], higher mistrust, β = 0.303, SE = 0.043, p < .001, 95% CI [0.219, 0.386], and greater credulity, β = 0.215, SE = 0.044, p < .001, 95% CI [0.129, 0.300]. Similarly to the BSI model, only mistrust and credulity significantly predicted PAI-BOR scores. Mistrust had a strong positive association with borderline traits, β = 0.274, SE = 0.046, p < .001, 95% CI [0.183, 0.365], and credulity also emerged as a significant predictor, β = 0.234, SE = 0.047, p < .001, 95% CI [0.141, 0.327]. Trust, however, was not a significant predictor of PAI-BOR scores, β = 0.013, SE = 0.036, p = .717, 95% CI [–0.057, 0.083]. These results reinforce the notion that ACEs shape epistemic stance and that mistrust and credulity serve as key psychological pathways linking early adversity to borderline personality features
